# Recognition of soybean pods and yield prediction based on improved deep learning model

**DOI:** 10.3389/fpls.2022.1096619

**Published:** 2023-01-13

**Authors:** Haotian He, Xiaodan Ma, Haiou Guan, Feiyi Wang, Panpan Shen

**Affiliations:** College of Information and Electrical Engineering, Heilongjiang Bayi Agricultural University, Da Qing, China

**Keywords:** soybean pods, deep learning model, pod recognition, phenotypic calculation, yield prediction model

## Abstract

As a leaf homologous organ, soybean pods are an essential factor in determining yield and quality of the grain. In this study, a recognition method of soybean pods and estimation of pods weight per plant were proposed based on improved YOLOv5 model. First, the YOLOv5 model was improved by using the coordinate attention (CA) module and the regression loss function of boundary box to detect and accurately count the pod targets on the living plants. Then, the prediction model was established to reliably estimate the yield of the whole soybean plant based on back propagation (BP) neural network with the topological structure of 5-120-1. Finally, compared with the traditional YOLOv5 model, the calculation and parameters of the proposed model were reduced by 17% and 7.6%, respectively. The results showed that the average precision (AP) value of the improved YOLOv5 model reached 91.7% with detection rate of 24.39 frames per millisecond. The mean square error (MSE) of the estimation for single pod weight was 0.00865, and the average coefficients of determination R^2^ between predicted and actual weight of a single pod was 0.945. The mean relative error (MRE) of the total weight estimation for all potted soybean plant was 0.122. The proposed method can provide technical support for not only the research and development of the pod’s real-time detection system, but also the intelligent breeding and yield estimation.

## 1 Introduction

Soybean is not only one of the five major crops in the world, but also an essential high protein grain and oil crop (Yu et al., 2022). As a leaf homologous organ (Lu et al., 2022), soybean pods are an important factor in determining grain yield and quality. Therefore, it is necessary to detect the pod’s quality of soybean plants in different growth stages and analyze the phenotypic characters of different varieties of pods. At the same time, it is also one of the important methods for identification and screening of soybean varieties (Zhao C. et al., 2021).

In recent years, the target detection technology based on deep learning had been applied to detect the traits of crop ecology and morphology, and had achieved good results in measuring and analyzing crop fruit, disease, stem, growth and yield estimation (Jia et al., 2021; Zhu et al., 2021; Lee and Shin, 2021; Sharma et al., 2022; Fu et al., 2022a). Kong et al. (2021) proposed a fruit target detection and positioning method based on Darknet depth framework YOLOv4, which realized the accurate positioning and recognition of the different kinds of fruits in the picture. Gao et al. (2022) used the Yolov4-micro network integrating with the channel spatial reliability discriminant correlation filter (CSR-DCF) to detect and count the apple fruit. Mirhaji et al. (2021) selected the yolov4 model to recognize the oranges on the fruit trees in the image, realizing the estimation of the orange yield in the orchard. Chen et al. (2022) used a new revolution bottleneck module and added an SE module on the basis of the original YOLOv5 network model to identify plant diseases under the natural conditions, accurately. Anam et al. (2021) trained the convolutional neural network (CNN) to segment the affected area of rice leaf disease based on the local threshold segmentation. Mathew and Mahesh (2022) used YOLOv5 network model to classify the acquired pictures of pepper leaf diseases, and realized the effective detection of bacterial spot disease on pepper leaves in the farm. Liu H. et al. (2020) built a CNN model with multi-scale hierarchical features based on the deep learning framework Tensor Flow, and realized the accurate identification of corn seedling stems. Ma et al. (2021) introduced the image augmentation technology in the original Mask R-CNN network layer to expand the image samples, and proposed an effective segmentation method of rice stem impurities based on improved Mask R-CNN. Fu et al. (2022b) used the YOLOv4 network model to realize the rapid real-time detection of banana bunches and stems in the natural environment. Zhou et al. (2021) used UAV images and convolution neural network to estimate the yield of soybean breeding varieties under drought stress. Yang et al. (2022) proposed a pod length and width calculation method based on Mask R-CNN network structure, which realized rapid segmentation of pods from pictures and effective calculation of the pod’s shape traits. Uzal et al. (2018) estimated the number of seeds per pod in plant breeding based on customized feature extraction (FE), support vector machine (SVM) and convolutional neural network (CNN). Yan et al. (2020) used five kinds of deep learning network models to identify one pod, two pods, three pods and four pods of mature soybean in the picture, accurately. Guo et al. (2021) improved the yolov4 target detection algorithm by integrating the K-means clustering algorithm and the attention mechanism module, and realized the detection of the number of pods per plant and the number of seeds in pods. Li et al. (2021) proposed a set of SPM-IS soybean phenotype measurement framework composed of the characteristic pyramid network, principal component analysis algorithm and instance segmentation network, which realized the effective measurement of the pod length, pod width, node length, main stem length, grain length, grain width, number of pods, number of nodes and number of nodes. Ning et al. (2021) proposed a phenotypic information extraction method for the soybean plant based on IM-SSD+ACO algorithm, which realized the extraction of soybean phenotypic traits including the number of pods, plant height, number of branches, main stem and plant type, effectively. Lu et al. (2022) proposed a method based on the YOLOv3 algorithm to predict soybean yield according to the number of pods and leaves. Zhang et al. (2021) constructed a soybean yield prediction model based on skew parameters using the color of soybean canopy leaves at different growth stages as input values.

At present, the recognition and counting of soybean pods as well as the detection of morphological and physiological phenotypes are not systematic, and there is a lack of methods for the recognition of soybean pods under natural growth and the estimation of pods weight per plant. In order to overcome the shortcomings of traditional artificial pods counting and yield estimation, such as time-consuming, error-prone and subjective factors, in this paper, a pod counting method based on improved YOLOv5 model was proposed. And then the pod phenotypic traits of pods were calculated including length, width, area, chord length and convex arc length. On this basis, the prediction model was established to reliably estimate the yield of the whole soybean plant based on Back Propagation (BP) neural network with the topological structure of 5-120-1.

## 2 Data acquisition and image preprocessing

### 2.1 Experimental materials

The cultivation of soybean plants and the acquisition of pods information were carried out in Heilongjiang Bayi Agricultural University of China. Soybean cultivation experiment was based on the agronomic background of exploring the changes in the ecological and morphological growth process of various organs of soybean under drought stress. The objective of this study was to establish a yield estimation method based on the number of soybean pods. The experiment was conducive to accurate control of soil, fertilizer, water and other environmental factors, reflecting the differences of plant characteristics under different growth stages. The cultivation experiment was carried out under the outdoor condition of 20~34°C, and the soybean varieties Suinong 26 and Heihe 49 were selected. During soybean planting, the medium sized soil block was paved on the bottom of the basin made of Polyvinyl chloride (PVC) with a diameter of 0.3m and a height of 0.18m, and then the screened non saline alkali fine soil was loaded until the basin weight was 5kg. After the compound fertilizer was paved evenly, the fine soil shall be put into the basin to 8.32kg. A total of 60 pots of two varieties were planted, with 6 holes in each pot and 2 soybeans in each hole. At the first trifoliolate stage (V1), 24 pots of single soybean and 36 pots of multi soybean were reserved.

### 2.2 Construction of image acquisition system

**Figure 1:**
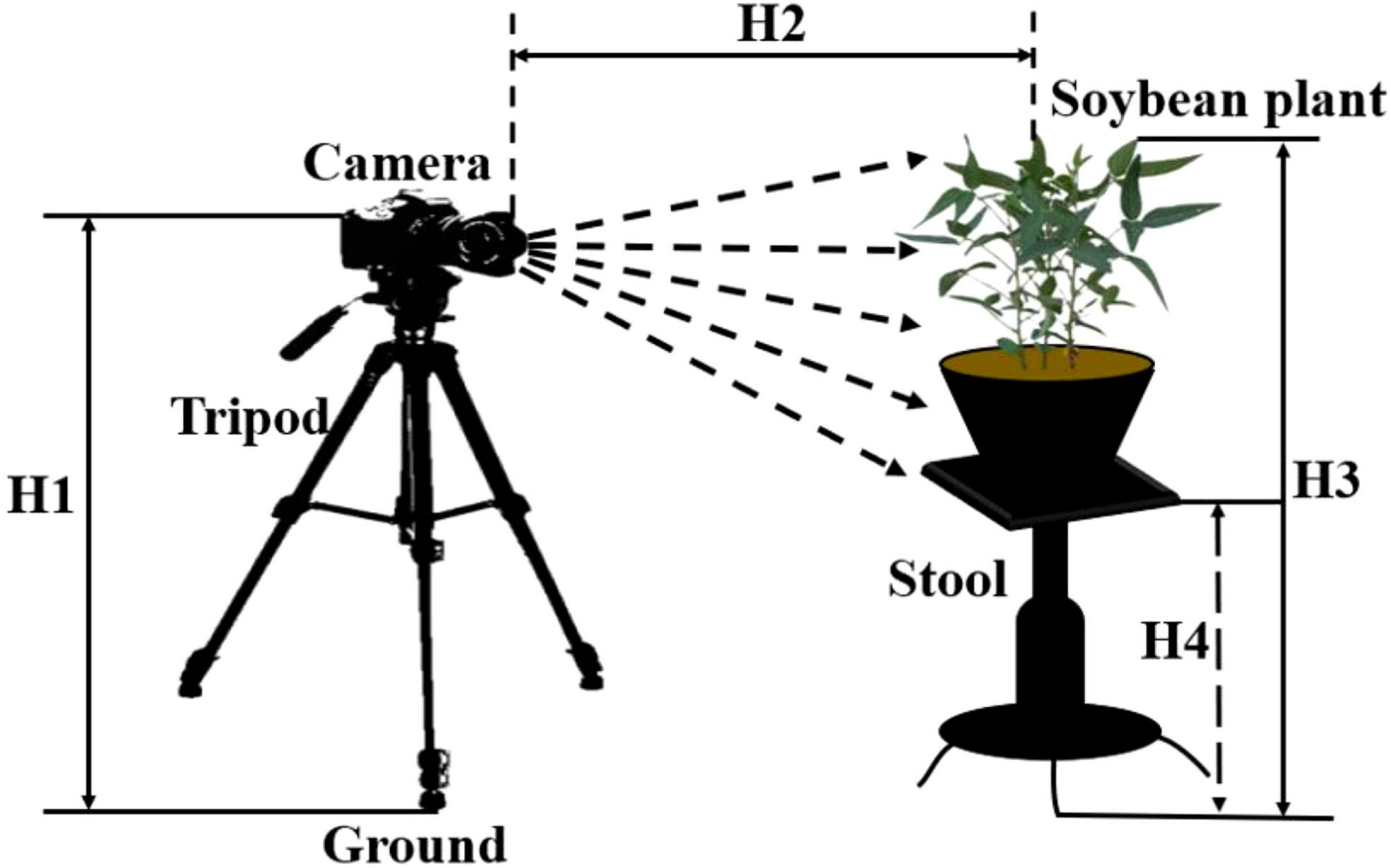
Schematic diagram of acquisition device for soybean plants images.

A soybean plants image acquisition system based on digital camera was constructed to dynamically acquire the digital image data of soybean plants in different growth stages. The growth stages of soybean studied included the beginning seed stage (R5), the full seed stage (R6), the beginning maturity stage (R7) and the full maturity stage (R8). The soybean plant image acquisition system device was shown in **Figure 1**. The acquisition system was composed of a digital camera, a lifting tripod support and a lifting platform. The acquisition platform used Canon 700D camera and Canon IS USM zoom lens, and the resolution of the captured image was 5184 pixels × 3456 pixels. The vertical distance H1 between the camera and the ground was 80 cm, and the horizontal distance H2 between the camera and the soybean plant was always kept at 100 cm. The vertical distance H4 was adjusted between the lifting platform and the ground according to the height of the soybean plant, so that the vertical distance H3 between the soybean plant and the ground was kept at 95 cm. In order to make the acquired the images of soybean plants in the natural growth state, the windless and sunny weather conditions were selected to complete the data acquisition.

### 2.3 Data acquisition method

**Figure 2:**
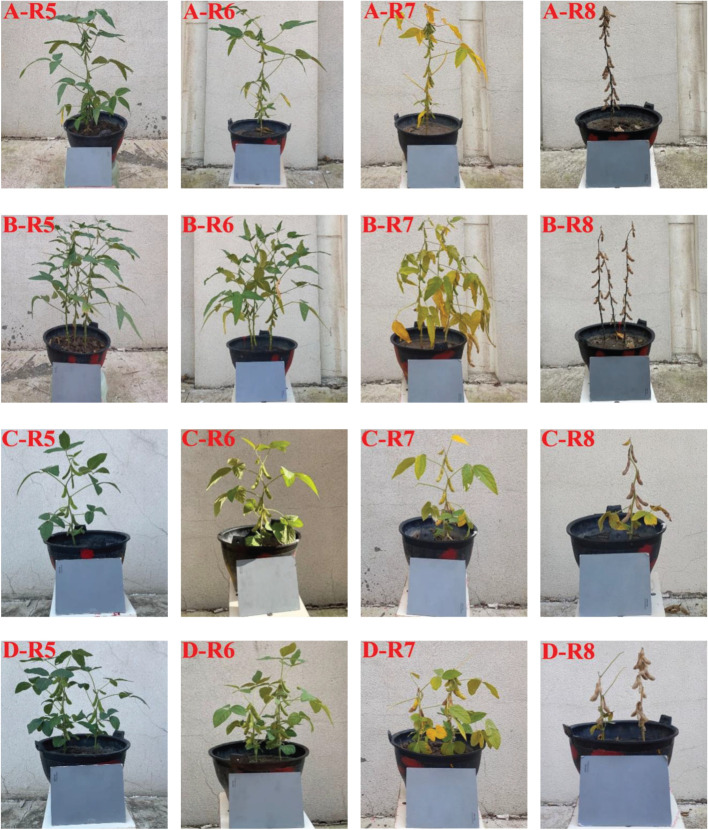
Soybean plants’ images of two varieties in different growth stages **(A)** Single soybean plant of Suinong 26 ; **(B)** Multi soybean plants of Suinong 26; **(C)** Single soybean plant of Heihe 49; **(D)** Multi soybean plants of Heihe 49.

For the integrity of experimental data, soybean plants of two varieties in different growth stages were acquired as experimental samples from 2020 to 2022. In order to avoid the overfitting phenomenon caused by the insufficient diversity of sample data, all soybean potted plants were rotated 360 ° for three times with the marked point as the starting position, and each rotation angle was 120 °. In this experiment, soybeans were collected from R5. According to the growth status, the data were collected every 4-10 days. A total of 863 digital images of soybean plants in different growth stages were acquired, including 216 at R5, 216 at R6, 216 at R7 and 215 at R8. **Figure 2** showed the images of soybean plants of two varieties in different growth stages.

### 2.4 Data annotations

**Figure 3:**
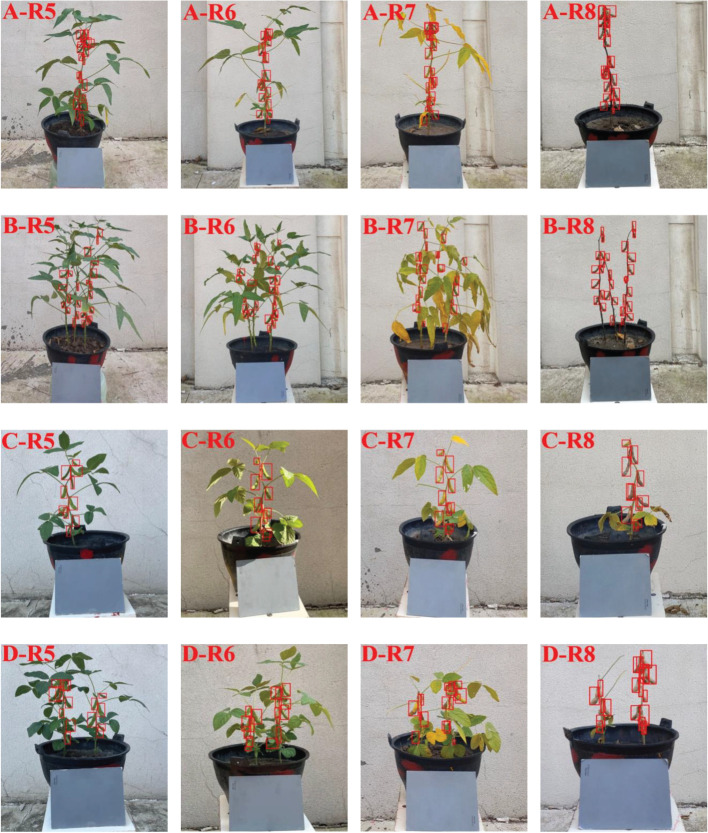
Soybean pod’s marking images of two varieties in different growth stages **(A)** Single soybean plant of Suinong 26; **(B)** Multi soybean plants of Suinong 26; **(C)** Single soybean plant of Heihe 49; **(D)** Multi soybean plants of Heihe 49.

In order to ensure the accuracy of the data and effectively train the detection model, it was necessary to manually label the data before data training. In this study, the LabelImg, an image annotation tool, was used to locate and mark the pods on 863 soybean plants images acquired in different growth stages. When labeling, the smallest circumscribed rectangle of the pods was taken as the real box, so as to reduce the useless pixels on the background in the box. The marked results were stored in .xml file, which contained information such as the position of the pods, the size of the anchor boxes and the label of the pods in the image. A total of 21650 pods were marked on 863 pictures in different growth stages. **Figure 3** showed the labeling of single and multiple soybean pods of two varieties in different growth stages.

### 2.5 Data augmentation and dataset partitioning

In this paper, according to the distribution characteristics of pods in soybean plants, the raw images were rotated, and then Gaussian noise was added and brightness was changed without changing the original data characteristics, so as to improve the detection accuracy and realize the robustness of the model.

After data augmentation of the original data, due to the impact of the quality of the raw image, some picture labels might exceed the pod’s calibration range or suffer serious quality loss. Therefore, it was necessary to manually select pictures. Finally, 322 poor quality pictures were removed, and 3130 pictures were obtained, including 863 raw images and 2267 enhanced images. In this paper, the raw images and the enhanced images were combined into a data set, including images and labels, a total of 6260 files. The images in all data sets were divided into training set, testing set and validation set according to ratio of 7:2:1. The specific distribution was shown in **Table 1**.

**Table 1:** Distribution of soybean plants in each data set in different growth stages.

Data set	Total number of images	The number of pictures in different growth stages			
		R5	R6	R7	R8
Training set	2191	550	549	549	543
Testing set	626	157	159	156	154
Validation set	313	83	78	77	75

## 3 Principle and method

### 3.1 Overall framework of pods’ recognition and yield prediction methods

**Figure 4:**
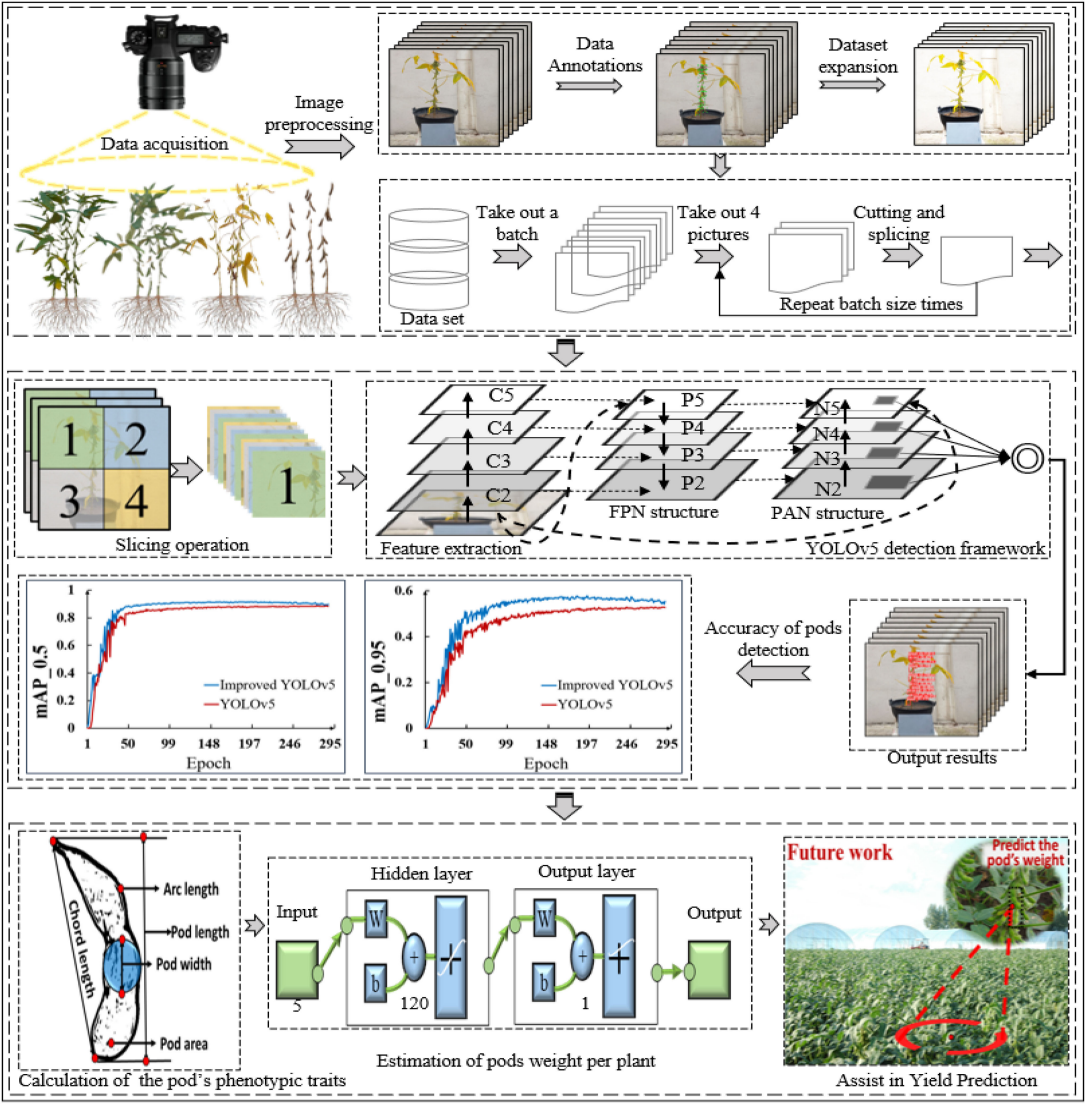
Overall framework of the soybean pod’s recognition and yield estimation.

First, the soybean plants data in different growth stages (R5~R8) were dynamically obtained by using the acquisition system of soybean plants and data augmentation and dataset expansion were carried out by using image processing algorithms, including random rotation, Gaussian filtering and adjusting image primary color. On this basis, the YOLOv5 model was improved by embedding CA module and using EIOU Loss instead of GIOU Loss as the regression loss function based on boundary box, so as to realize the recognition and counting of soybean pods in different growth stages. Then, the pod’s length, width, area, chord length and convex arc length were calculated by using the minimum circumscribed rectangle method, the maximum inscribed circle method, the regional pixel counting and template calibration method, and the combination of contour convex hull and endpoint detection method. The validity of the pod’s phenotype calculation method was verified by establishing the linear correlation between measured and calculated values. Finally, the weight estimation model of single pod was constructed based on BP neural network with topological structure of 5-120-1. The average weight of a single pod of two varieties was estimated. Combined with the number of pods per plant identified by the improved YOLOv5 algorithm, the weight of the whole plants pods was estimated. **Figure 4** showed the overall framework of the soybean pod’s recognition and estimation of all pods weight per plant.

### 3.2 Recognition method of the pod based on improved YOLOv5 algorithm

#### 3.2.1 YOLOv5 network model

YOLOv5 network model was mainly divided into four parts: input end, backbone network, characteristic network architecture and output end (Gu et al., 2022). The input end was mainly used to preprocess the image. Mosaic data augmentation operation (Zhao B. et al., 2021), adaptive anchor box calculation (Gao et al., 2019) and adaptive image scaling were used to scale the input image to the input size of the network, and normalization was performed at the same time. The backbone network structure mainly included the Focus module that sliced the images, the Bottleneck Cross Stage Partial (CSP) module and the Spatial Pyramid Pooling (SPP) module were used to fix the image size. Feature network architecture mainly solved the problem of multi-scale detection in target detection. SPP module and FPN+PAN module located in the middle of backbone network and input terminal were used to further improve the diversity and robustness of target features. The output included a classification branch and a regression branch, which were mainly used to output the target recognition results.

#### 3.2.2 Embedding CA attention mechanism

**Figure 5:**
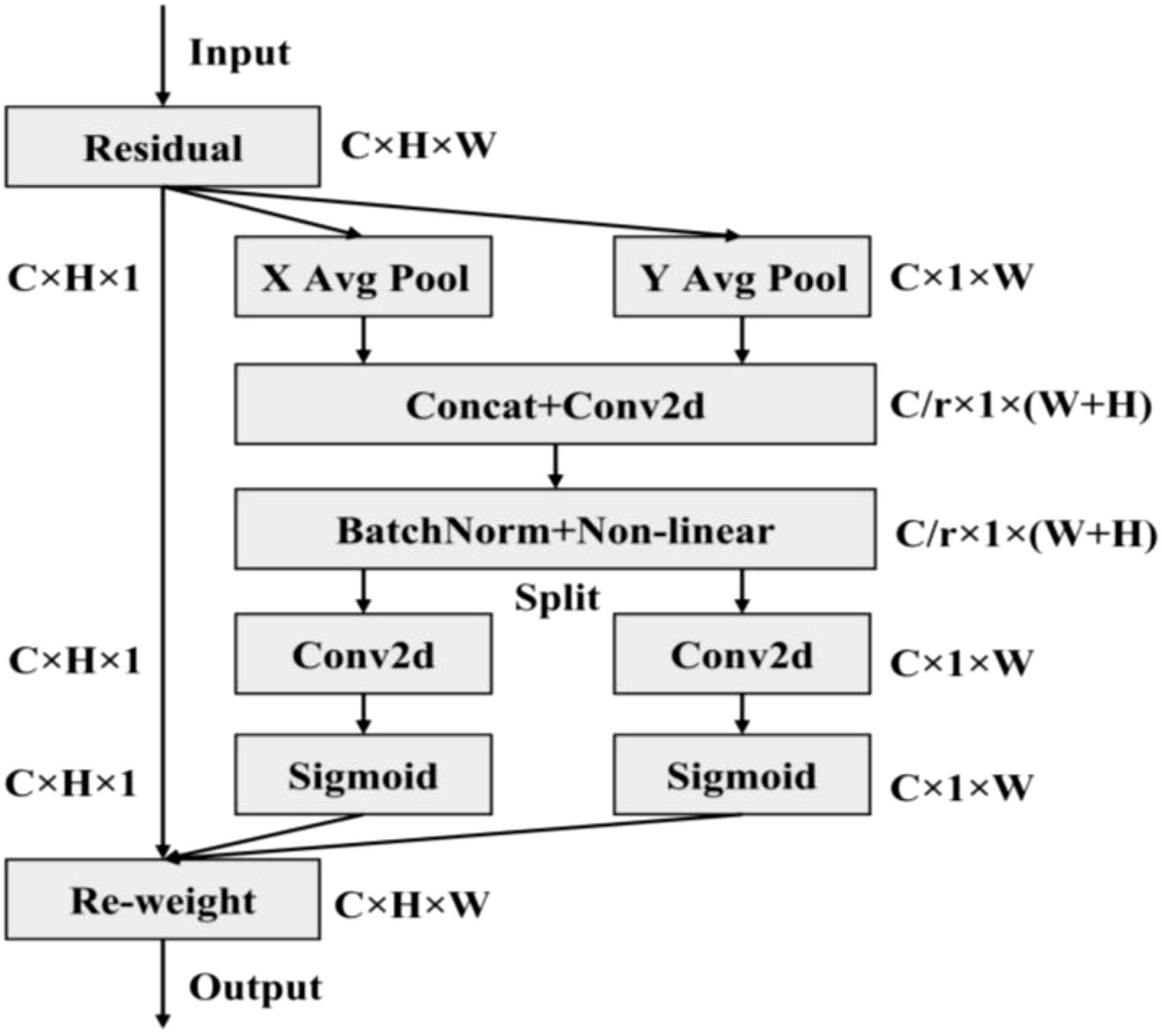
Structure of the CA module.

Attention mechanism mimics biological vision and mainly scans the whole image quickly to screen out the regions of interest, and invests more attention resources to suppress other useless information and improves the efficiency and accuracy of visual information processing (Qi et al., 2022). Because the soybean pods target was small and easy to be disturbed by background factors, YOLOv5 model was easy to lose the characteristic information of small targets during convolution sampling, resulting in missed detection and false detection. Therefore, the CA module (Xu X. et al., 2022) was embedded in YOLOv5 model. Through the channel attention module and spatial attention module, the weight of small targets in the whole feature map could be increased effectively. The effective extraction of the target pod’s feature information was realized, and the accuracy of soybean pods’ recognition was improved in different growth stages. **Figure 5** showed the structure of CA module.

First, the global pooling operation was performed in Channel Attention Module on the spatial dimension of the input characteristic graph with the size of C×H×W. The size of feature map after operation was C×H×1. The eigenvalues obtained by the average pooling operation mainly described the background information of the image, and the eigenvalues obtained by the maximum pooling operation mainly described the texture information of the image. Then, the pooled results were sent to the two shared parameters of neural networks respectively. After spliced in the channel dimensions, the two groups of pooled output results were multiplied and added separately, and the weight range was constrained to the (0,1) interval with the help of the activation function. Finally, the input feature map was weighted to obtain the channel attention feature map, so as to enhance the expression of pod information, suppress the expression of useless background information, and improve the recognition effect. The output channel representation of the height and width of the target box was as follows:


(1) $$Z_{\text{c}}^{h}(h)=\frac{1}{w}\sum_{0<i\leq w}X_{c}(h,i)$$

(2) $$Z_{\text{c}}^{w}(w)=\frac{1}{H}\sum_{0<i\leq H}X_{c}(j,w)$$

Where, $Z_{c}^{h}$ represented the output of the *c* channel with a height of *h*, and $Z_{c}^{w}$ represented the output of the c channel with a width of w. Equation (1) and (2) aggregated features along two spatial directions, returning a pair of directional perceptual attention features $Z_{c}^{h}$ and $Z_{c}^{w}$ , and generated a pair of feature maps at the same time, making the target location to be detected more accurate.

Different from the channel attention module, the spatial attention module extracted features through the spatial dimension information of the feature map. First, the average pooling and maximum pooling operations were performed in the spatial attention module on the channel dimension for the input characteristic graph of size C×H×W. The size of the two feature maps after operation was 1, which reduced the increase of parameters. The two one-dimensional feature maps were spliced into a two-dimensional feature map based on channel dimensions. Then, 7×7 convolution layers were used to extract the mask map that described the location information of the feature space in the feature map. After constrained by the activation function, the mask map was applied to the input feature map to obtain the spatial attention feature map enhanced according to the spatial location information, so as to improve the expression of pod shape, size, color, texture and other features. The specific Equation was as follows:


(3) $$f=\delta (F_{1}(\left[z^{h},z^{w}\right]))$$

(4) $$g^{h}=\sigma (F_{h}(f^{h}))$$

(5) $$g^{w}=\sigma (F_{w}(f^{w}))$$

(6) $$y_{c}(i,j)=x_{c}(i,j)\times g_{c}^{w}(j)$$

Where, *F*_1_ represented 1*1 convolution, *f* represented the intermediate feature image obtained through the down sampling operation *δ*, and two separate tensors *f^h^* and *f^w^* could be obtained after segmentation along the spatial dimension. Then, *g^h^* and *g^w^* with the same channel number as the input image *X* could be obtained by 1*1 convolution *F_h_*, *F_w_* and σ transformation. After expansion, they were added to the input as attention weights, and *y* represented the final output image.

**Figure 6:**
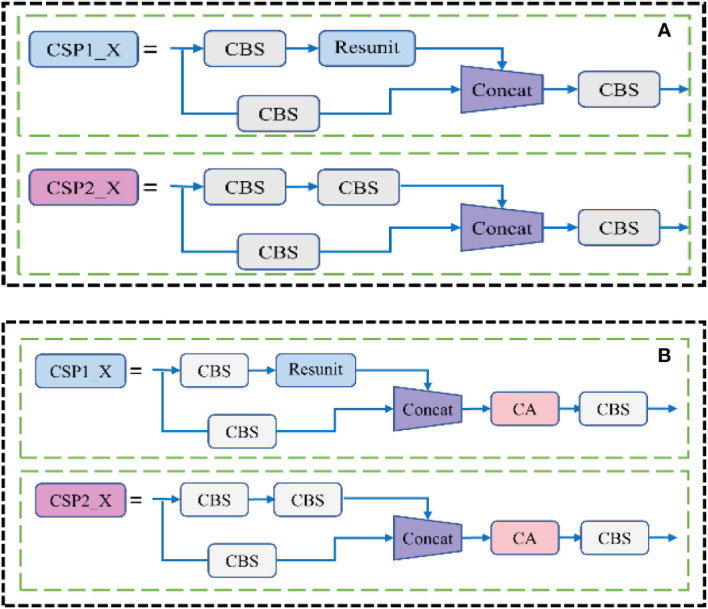
Model comparison before and after the CA module was embedded in the CSP structure **(A)** Before improvement; **(B)** After improvement.

The CSP module before and after embedded the attention mechanism was shown in **Figure 6**. The CA module decomposed the channel attention into two one-dimensional features along different spatial directions for coding, which could not only capture the long-range dependence along one spatial direction, but also saved the accurate location information along the other direction, while expanding the global receptive field of the network. This method could not only locate the pod’s target more accurately, but also saved a lot of computing overhead.

#### 3.2.3 Improved border regression loss function

Loss function was one of the important criteria to judge whether a model was applicable to the current data set. This function was used to characterize the fitting degree between the predicted value and the real value. When the loss function curve gradually converged, the model had achieved a relatively ideal prediction effect (Lv and Lu, 2021). Yolov5 used GIOU Loss as the loss function of the bounding box (Wang H. et al., 2022), and used binary cross entropy and Logits loss function to calculate the loss of class probability and target score. The calculation Equations were as follows:


(7) $$Loss_{coord}=\sum_{i=0}^{S^{2}}\sum_{j=0}^{B}1_{ij}^{obj}(1-GIOU_{ij})$$

(8) $$GIOU_{ij}=\frac{J}{U}-\frac{A-U}{A}$$

(9) $$U=\hat{\omega_{i}}\cdot \hat{h_{i}}+\omega_{i}\cdot h_{i}-J$$

(10) $$IOU=\frac{A\cap B}{A\cup B}$$

Where, *Loss_coord_* represented the loss function of the target location, *S* represented the grid of S ×S each containing the prediction results, *B* represented two prediction boxes, $1_{ij}^{obj}$ represented the target contained in the prior box *j* generated by cell *i*, *J* represented the intersection area of the border, *U* represented the union area of the border, *A* represented the minimum circumscribed rectangular area of the border, *ω_i_* and *h_i_* represented the length and width of the prediction box, respectively, IOU represented the ratio of intersection and union between prediction frame and real frame. When the prediction frame coincided with the real frame, the maximum GIOU was 1. On the contrary, as the distance between the prediction frame and the real frame increased, GIOU tended to be -1, that was, the farther the distance between the prediction frame and the real frame was, the greater the loss value was. When the distance between the prediction frame and the real frame was inclusive or the width and height were aligned, and the difference set was 0, the loss function could not be derived and could not converge, which was easy to cause false detection and missed detection for the pods covered by leaves or stems. Therefore, in this study, the EIOU Loss frame regression loss function was used instead of the GIOU Loss frame loss function as the deviation index of the prediction frame deviation (Fan et al., 2022). EIOU Loss function included three parts: overlap loss, center distance loss and width height loss. The width height loss directly minimized the difference between the width and height of the target box and the anchor box. EIOU Loss was shown in Equation (11):


(11) $$L_{EIOU}=L_{IOU}+L_{asp}=1-IOU+\frac{\rho^{2}(b,b^{gt})}{c^{2}}+\frac{\rho^{2}(w,w^{gt})}{c_{w}^{2}}+\frac{\rho^{2}(h,h^{gt})}{c_{h}^{2}}$$

Where, *b* was the center point of the prediction frame, *b^gt^* was the center point of the real frame, *ρ* was the Euclidean distance between the two center points, *c* was the diagonal distance of the smallest closure area that can contain both the prediction frame and the real frame, *c_w_* and *c_h_* were the width and height of the minimum circumscribed rectangle box covering the prediction frame, respectively, *w* and *w^gt^* represented the width of the prediction box and the real box, respectively.

In the boundary box regression loss function, EIOU Loss avoided the non- convergence when the real box and the prediction box were in the inclusion relationship, and could improve the recognition accuracy of pods in the case of occlusion effectively. Its width and height loss made the convergence speed faster and the accuracy higher, and its performance was better than that of the traditional YOLOv5 boundary loss function.

### 3.3 Estimation of soybean yield based on multi-dimensional pod’s phenotypic traits

#### 3.3.1 Calculation method of pod’s phenotypic traits

**Figure 7:**
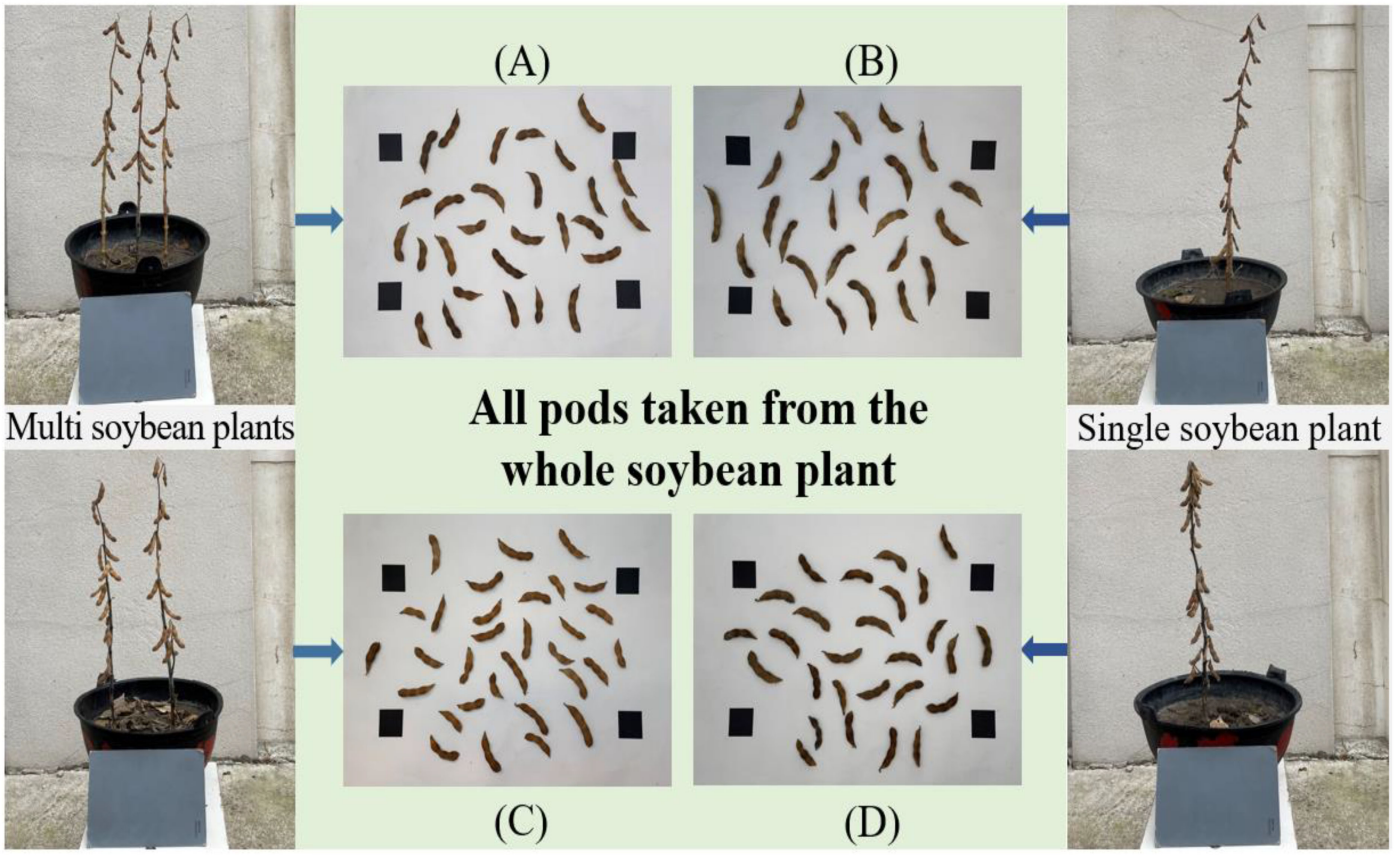
All mature soybean pods picked **(A)**, **(C)** Pods on multiple soybean plants; **(B)**, **(D)** Pods on single soybean plant.

The yield of soybean crops was closely related to the number of pods per plant, the phenotypic characters of pods and the degree of plumpness of pods. Therefore, it was of great significance to quickly and accurately obtain the pod’s physiological and ecological indicators that determined the grain yield and quality for cultivating soybean varieties with high yield and quality traits and estimating the yield. In this paper, when the soybean plant grew to full maturity, all the pods on the mature soybean plants of the two varieties were picked, and the phenotypic traits including length, width, area, chord length and arc length were automatically calculated (He et al., 2022). **Figure 7** showed pods picked from selected soybean plants randomly.

**Figure 8:**
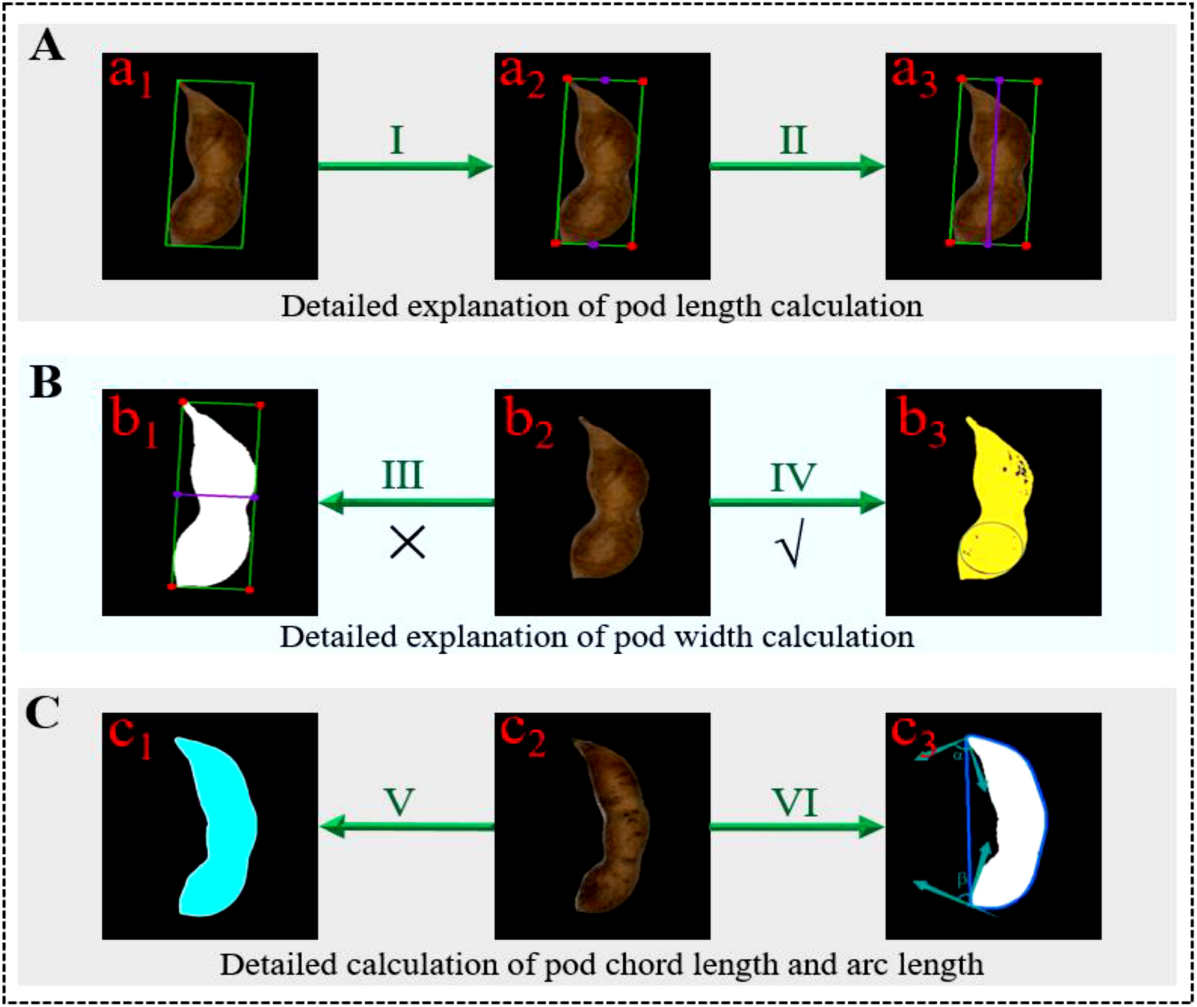
Steps for calculating phenotypic traits of a single pod **(A)** Calculation of pod length; **(B)** Calculation of the pod width; **(C)** Calculation of pod area, chord length and arc length.

In order to calculate the phenotypic traits of soybean pods more accurately, it was necessary to carry out geometric distortion correction, Gaussian filter noise reduction (Wang G. et al., 2022), Canny edge detection operator (Luo et al., 2021) and morphological close operation to extract the pod’s contour and other preprocessing on the pod’s images. It provided a reliable data base for the calculation of phenotypic traits of soybean pods. **Figure 8** was the schematic diagram for calculating the phenotypic traits of a single pod.

##### 3.3.1.1 (1) Calculation of the pod length

In this study, the minimum circumscribed rectangle algorithm was used to determine the circumscribed rectangle of a single pod. The length of the long side of the circumscribed rectangle was taken as the length of the pods. The calculation ratio of the pods in the image was defined by the black marker blocks with length and width of two centimeters, and the actual size of the pods in the image was calculated by combining the Euclidean distance Equation. The specific definitions were as follows:


(12) $$U=\frac{K_{\alpha }}{K_{\beta }}$$

(13) $$D=\sqrt{{\left(x_{2}-x_{1}\right)}^{2}+{\left(y_{2}-y_{1}\right)}^{2}}$$

Where, *U* represented the calculation ratio of the object size, *K_α_* represented the pixel length of the object, and *K_β_* represented the real length of the object. *D* represented the Euclidean distance between points (*x*_1_,*y*_1_) and (*x*_2_,*y*_2_). In **Figure 8**, A represented the decomposition diagram of pod length calculation, a_1_ represented the schematic diagram of the minimum circumscribed rectangle of the pods, I represented the calculation of the vertex and the center points of the upper and lower sides of the rectangle, a_2_ represented the drawing of the endpoint and midpoint of the rectangle, II represented the calculation of the Euclidean distance between the upper and lower center points of the rectangle, and a_3_ represented the drawn centerline of the rectangle.

##### 3.3.1.2 (2) Calculation of the pod width

According to the definition standard of pod width in the specification for the description of soybean germplasm resources, the widest part of the pods was taken as the pod width. Therefore, the maximum inscribed circle algorithm (Huang et al., 2021) was used to determine the maximum width of the pods by finding and calculating the maximum inscribed circle diameter of the pods contour. The Equations of maximum inscribed circle center and radius were as follows:


(14) $$S=\frac{\sum_{i}x_{i}+x_{i-1})(y_{i}-y_{i-1})}{2}$$

(15) $$m=\frac{{\sum_{i}\left(x_{i}+x_{i-1}\right)}^{2}(y_{i}-y_{i-1})}{S}$$

(16) $$n=\frac{{\sum_{i}\left(y_{i}+y_{i-1}\right)}^{2}(x_{i}-x_{i-1})}{S}$$

(17) $$D=2R=2\sqrt{{\left(x_{i}-m\right)}^{2}+{\left(y_{i}-n\right)}^{2}}$$

Where, *s* was the area of the closed boundary contour, *x_i_* was the abscissa on the closed boundary contour, *y_i_* was the ordinate of the closed boundary contour, *m* was the abscissa of the center of the circle, *n* was the ordinate of the center of the circle, *R* was the radius of the maximum inscribed circle of the pods, and *D* was the maximum width of the pods. In **Figure 8**, B represented the schematic diagram of pod width calculation, in which III represented the common wrong calculation method of pod width, that was, it was considered that the width of the minimum circumscribed rectangle of the pods was the pod’s width, and b_1_ was its schematic diagram. IV represented the correct calculation method of pod width used in this paper, b_2_ was the raw diagram of pod, and b_3_ was the schematic diagram of the maximum width of pod.

##### 3.3.1.3 (3) Calculation of the pod area

In this study, the extracted pod’s contour was expanded and filled smoothly on the premise of clear image boundary. Then the pod area was calculated by combining the regional pixel counting and the template calibration method. Finally, the actual area of the standard reference block in the image was used to calculate the area of the pods. The calculation Equation was as follows:


(18) $$S_{\text{d}}=\frac{W_{d}\times S_{k}}{W_{k}}$$

Where, *S_d_* represented the actual area of the pods, *W_d_* represented the number of pixels of the pods, *S_k_* represented the actual area of the standard marker block, and *W_k_* represented the number of pixels of the standard marker block. In **Figure 8C**, V represented the calculation of pod area, c_1_ represented the calculation diagram of pod area, and c_2_ represented the raw diagram of pod.

##### 3.3.1.4 (4) Calculation of the chord and arc length

At present, the ratio of chord to arc was an index used by some breeders to describe the bending degree of the pods. The greater the ratio of chord to arc, the straighter the pod’s shape, the smaller the ratio, the more curved the pods. In this paper, first, the perimeter of the soybean pods contour was counted, and the polygon contour of the soybean pods was obtained by using the convex hull algorithm (Liu et al., 2018). Then, starting from the middle point on the left side of the contour, the vector angles of the two points close to the contour were calculated, and the two angles with the largest difference were obtained by dislocation subtraction. The two corresponding points were the two endpoints of the pod contour. In combination with Equation (13), the Euclidean distance between the two endpoints was the pod’s chord length. By making a difference between the perimeter of the external polygon of the pods and the chord length of the pods contour, the convex arc length of the pods was obtained. The vector angles of the two adjacent points of the pods were as follows:


(19) $$\cos\alpha =\frac{\left(x_{1}y_{1}+x_{2}y_{2}\right)}{\sqrt{\left(x_{1}^{2}+y_{1}^{2})(x_{2}^{2}+y_{2}^{2}\right)}}$$

Where, cosα was the cosine of the tangent vector of the two adjacent points of the pods contour, *x*_1_ and *x*_2_ were the abscissa of the two adjacent points of the pods contour respectively, *y*_1_ and *y*_2_ were the ordinates of the two adjacent points of the pods contour respectively. **Figure 8** VI showed the calculation of pod chord length and arc length, and c3 was its schematic diagram, α, β represented the included angle between the two ends of the pod and the adjacent points.

#### 3.3.2 Estimation method of soybean yield

**Figure 9:**
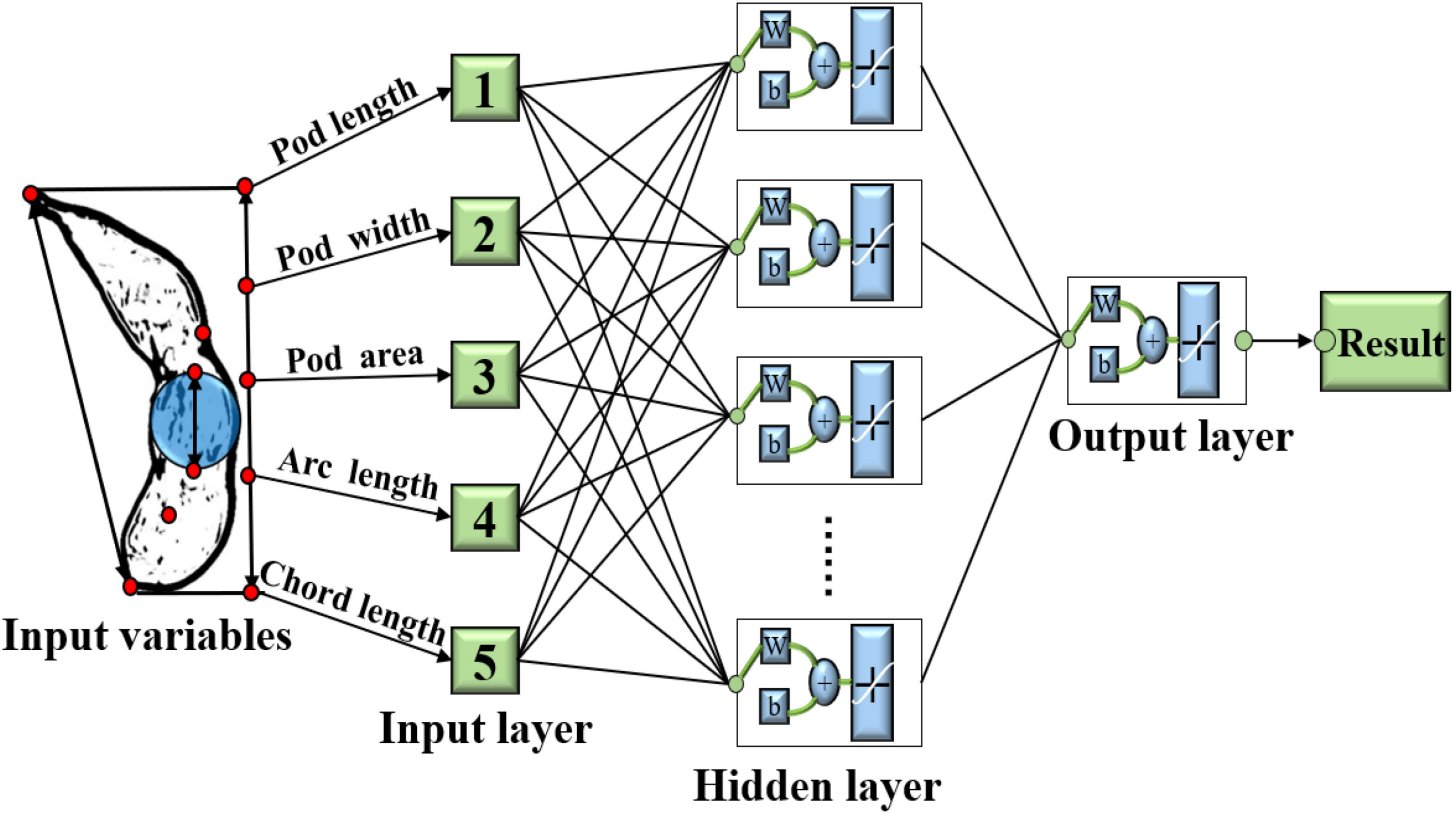
Structure diagram of neural network for estimation of single pod weight.

Because the soybean yield had the characteristics of randomness and nonlinearity, the accurate mathematical model could not predict the soybean yield, effectively. Therefore, in this study, a three-layer nonlinear BP neural network (Jiang et al., 2021) system was used to predict soybean yield. Due to the strong self-adaptive resolution performance and fault tolerance, BP network could approximate continuous nonlinear functions with arbitrary accuracy and had significant local approximation characteristics (Guan et al., 2013; Roopali and Toran, 2020), which provided a technical guarantee for accurate and efficient prediction of soybean yield. The pod’s phenotypic traits were the dimension of the feature space, which determined the number of nodes in the input layer. In this study, five traits including pod length, pod width, pod area, chord length and arc length were selected as the input values of the neural network, so the number of nodes in the network input layer was 5. The number of nodes in the output layer was determined according to the dimension of the mode space. Because the prediction result of pod weight was a specific value, the number of output nodes was 1. According to the theorem *Kolmogorov* (Liu M. et al., 2020) and practical experience, the number of neuron nodes in the hidden layer was determined to 120. Finally, the BP network topology for predicting the weight of the pod was 5-120-1 type, and each layer was fully interconnected (**Figure 9**). In order to reduce the training time and complete the training task efficiently, the iteration accuracy of training target was defined as 0.01, which was the termination condition of model training. The initial learning rate was set to 0.01 and gradually increased. The optimal learning rate was finally determined to 0.8 based on the value of training cost.

## 4 Results and analysis

### 4.1 Analysis of pods recognition results

#### 4.1.1 Training of pods recognition model

##### 4.1.1.1 (1) Setting environment parameters

This study used pytorch 1.9.0 machine learning framework, and the graphics processing unit (GPU) used NVIDIA GeForce GTX 1050 Ti (4096MB). Soybean pods recognition models in different growth stages were trained using YOLOv5 and improved YOLOv5 models on Windows 10 64-bit operating system. The input size of the images had a great impact on the performance of the detection model. Because a feature map ten times smaller than the raw images will be generated in the basic network, the details of smaller pods were not easy to capture. Thus, the input size of the image was adjusted to 640 × 640 (pixels) for training, which can improve the robustness of the detection model to the object size to a certain extent. In addition, the initial learning rate of the model, the size of the super parameter and the attenuation coefficient as well as the training round would affect the convergence of the loss function, thus affecting the accuracy of the model training. According to the parameter setting method proposed in the literature (Li. et al., 2022; Qiu et al., 2022), the initial learning rate was 0.001, the cosine annealing super parameter was 0.12, and the attenuation coefficient was 0.00036. A total of 300 epochs were trained.

##### 4.1.1.2 (2) Model training

**Figure 10:**
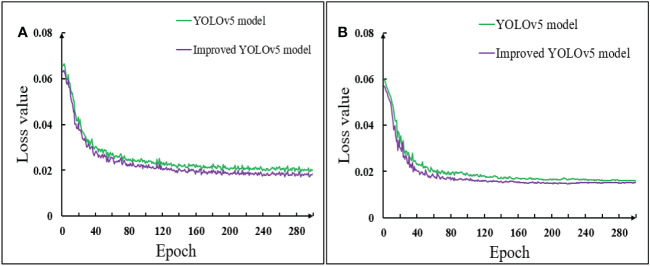
Comparison of loss function of model before and after improvement **(A)** Training set; **(B)** Validation set.

Loss function was one of the important criteria to measure the prediction effect of a model on the current data set. It mainly mapped the values of relevant random variables to non-negative real numbers to represent the gap between the prediction results and the measured data. When the loss function curve converged gradually, the model had achieved an ideal prediction effect. The comparison of the loss function changed between the training set and the validation set of the YOLOv5 model and the improved YOLOv5 model was shown in **Figure 10**.

**Figure 10** showed that during the pod’s recognition in the training set and the validation set, the early loss function of the model decreased rapidly. With the increase of the number of training rounds, the loss curve gradually decreased and tended to be stable. The loss value of the first 60 training groups in the training set decreased rapidly. When the epoch reached about 140, the loss value of the algorithm decreased to be stable, and the loss function value stabilized at about 0.02. The loss value of the first 40 training groups in the validation set decreased rapidly. When the epoch reached about 60, the loss value of the algorithm decreased to be stable, and the stable value of the loss function was also about 0.02. It could also be seen from the image that the loss curve of the improved YOLOv5 model of the training set and the validation set was always below the YOLOv5 model. It also showed that the loss value of the improved YOLOv5 model was always smaller than that of the YOLOv5 model, that was, the positioning accuracy was higher, the convergence speed was faster, and the prediction effect was better when identifying soybean pods in different growth stages.

#### 4.1.2 Prediction effect analysis of the recognition model

##### 4.1.2.1 (1) Evaluation of recognition effect of pods before and after improvement

**Figure 11:**
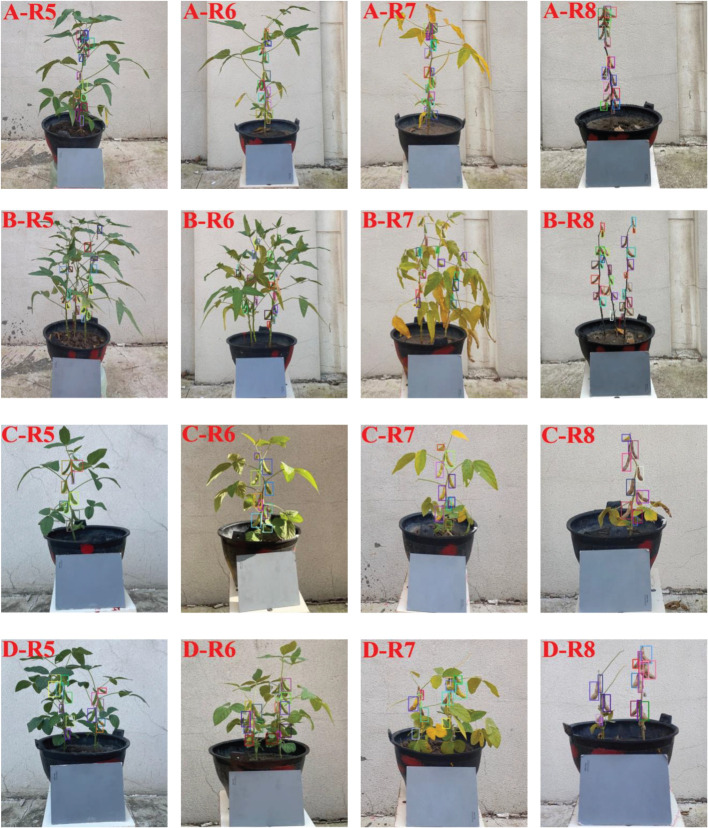
Recognition results of pods by using improved YOLOv5 model. **(A)** Single soybean plant of Suinong 26; **(B)** Multi soybean plants of Suinong 26; **(C)** Single soybean plant of Heihe 49; **(D)** Multi soybean plants of Heihe 49.

The prediction result of the model was the most intuitive way to evaluate the quality of a model. The pods in the testing set were recognized using the original model and the improved yolov5 model, which were shown in **Figure 11**.

**Figure 11**showed that for different varieties, Heihe 49 had better overall detection effect on soybean pod’s than Suinong 26. For the same variety, the recognition effect of single plant pods was better than that of multiple plants pods. For example, A-R5 and B-R5, there were 23 pods in A-R5, 22 of which were recognized correctly, with a recognition accuracy rate of 96%. There were 23 pods in B-R5, 20 of which were identified correctly, with a recognition accuracy rate of 87%. For different stages of the same variety, the recognition performance of the model was gradually enhanced with the maturity of soybean pods. In D-R7 and D-R8, there were 17 pods in D-R7, 14 of which were correctly recognized, with an accuracy rate of 82.4%. There were 20 pods in D-R8, 17 of which were correctly recognized, with an accuracy rate of 85%. This was because the learning performance of the model was affected by plants growth, leaves shading and pod’s maturity. The denser the leaves of soybean plants, the more serious the shielding between stems and pods, pods and pods, and between leaves and pods, and the worse the recognition effect of the model will be. As the pods mature, the greater the color contrast between pods and plant leaves and stems, the better the recognition effect of the model will be.

The prediction effect of different models was analyzed by comparing the number of Ture Positive (TP) and False Positive (FP). The calculation method for the number of TP and FP was to first obtain the real box and the prediction box after recognition of the pods. The prediction box contained the detection category, confidence score and coordinate information of the detection box. If the retention confidence score of the prediction box was greater than 0.3, the maximum matching IOU value was calculated between the prediction box and the real box. If the IOU value was greater than 0.5 and the two boxes were matched for the first time, the result was recorded as TP, otherwise it was recorded as FP. The more the number of TP, the higher the detection accuracy of the model, and the stronger the performance will become. The more the number of FP, the lower the detection accuracy of the model, and the worse the performance will become. The number of TP and FP of the original model and the improved YOLOv5 model in the test set was counted, which was shown in **Table 2**.

**Table 2:** Comparison of TP and FP between YOLOv5 model and improved YOLOv5model.

Model	TP/FP	Growth stage of soybean plants			
		R5	R6	R7	R8
YOLOv5	TP	1708	1725	1707	1697
	FP	504	378	336	227
Improved YOLOv5	TP	1795	1851	1729	1704
	FP	336	297	266	206

**Table 2** showed that, for the number of TP, the improved YOLOv5 model had significantly more TP detected for soybean pods in different growth stages than the YOLOv5 model. The number of TP at R5-R8 was 87, 126, 22 and 7 more than that in the original model, respectively. For the number of FP, the number of FP detected by the improved YOLOv5 model for soybean pods in different growth stages was significantly less than that of the YOLOv5 model, in which the maximum difference in the number of FP in R5 was 168, and the minimum difference in the number of FP in R8 was 21. It could also be seen from the table that the number of FP was the largest in R5. With the growth of soybean plants, the number of FP detected by the model was gradually decreasing. The main reason for this situation was that the soybean plants had dense leaves at R5 and R6 stages, and the leaves and stems had a serious shelter against the pods, and the morphology of some new leaves was very similar to that of the pods, resulting in the error of model recognition. At R7 and R8 stages of soybean plants, with the falling off of leaves and stems, the blocking phenomenon gradually disappeared, so the error rate of pod’s recognition decreased.

**Figure 12:**
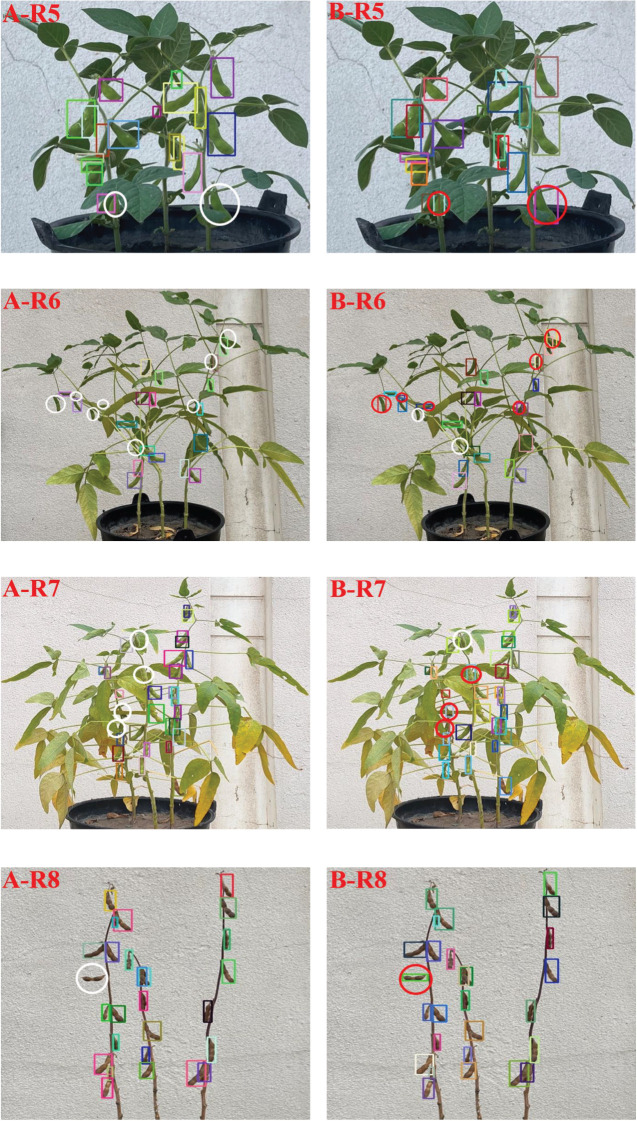
Comparison of partial recognition results before and after model improvement **(A)** YOLOv5 model; **(B)** Improved YOLOv5 model.

In order to more intuitively compare the difference between the detection results before and after the model improvement, a pot of soybeans in each of the four stages identified and output by YOLOv5 and the improved YOLOv5 algorithm was randomly selected in the testing set. The difference between the recognition results of the two models was shown in **Figure 12**.

**Figure 12** showed that all the pods not detected in the YOLOv5 model of soybean plants at R5 and R8 stages were recognized in the improved YOLOv5 model. At R6 stage, 8 pods were not recognized in the YOLOv5 model and 6 more were recognized in the improved YOLOv5 model, but 2 pods were still not recognized because the leaves were too dense and covered by the stems. At R7 stage, 4 pods were not recognized in the YOLOv5 model, 3 more were recognized in the improved YOLOv5 model, and 1 pod was not recognized because its shape and color were very similar to soybean leaves. For the situation that the YOLOv5 model had missed detection, the improved YOLOv5 model had been significantly improved. This result showed that the improvement of YOLOv5 model in this study was effective for the recognition of soybean pods in different growth stages.

##### 4.1.2.2 (2) P-R curve analysis of model prediction before and after improvement

**Figure 13:**
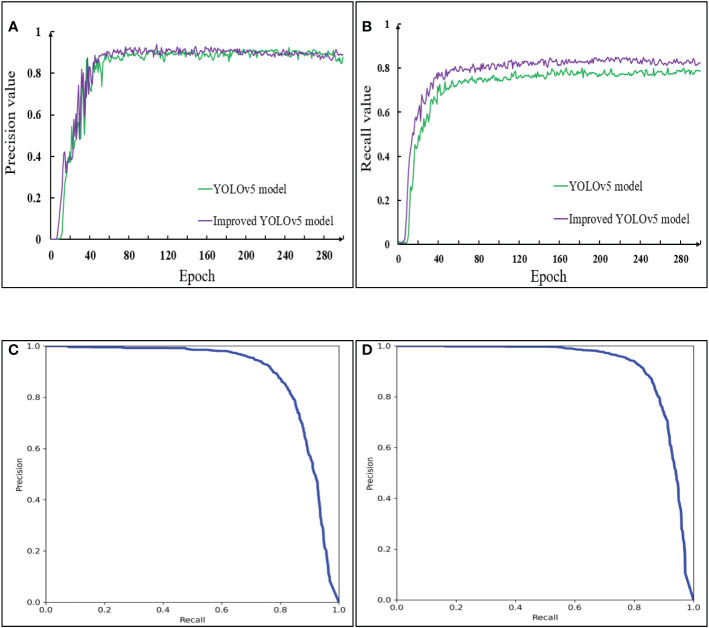
Comparison of P-R curve between YOLOv5 model and improved YOLOv5 model in the testing set. **(A)** Precision; **(B)** Recall; **(C)** P-R curve of the YOLOv5; **(D)** P-R curve of the Improved YOLOv5.


The precision (P), recall (R) and P-R curve (Wang Z. et al., 2022) of pods recognition were compared between YOLOv5 and the improved YOLOv5 model on the testing set (**Figure 13**). The precision and recall were a pair of contradictory variables, the higher the precision, the lower the recall. In order to balance the relationship between the two indexes, was the area under the P-R curve was used as the average precision value to evaluate the performance of the model. The specific Equation was as follows:

(20) $$P=\frac{TP}{TP+FP}\times 100\%$$

(21) $$R=\frac{TP}{TP+FN}\times 100\%$$

Where, *P* and *R* represented the precision rate and recall rate respectively, TP(True Positive) represented that the positive sample was judged as a positive sample, FP(False Positive) represented that the negative sample was judged as a positive sample, and FN(False Negative) represented that the positive sample was judged as a negative sample. The average precision AP value of pods recognition could be obtained by calculating the area of the lower part in the P-R curve coordinate system. Since this article only contained one class of identification targets, the AP value was the mAP value of all classes. The Equation was as follows:


(22) $$AP=\int_{0}^{1}PRds$$

(23) $$mAP=\frac{1}{N}\sum_{m=1}^{N}AP_{m}$$

Where, *AP* represented the area below the P-R curve, *N* represented the total number of categories. *mAP* was the result of averaging the *AP* values of all prediction categories. The larger the mAP value was, the better the prediction effect of the model was.

**Figure 13A** showed that the precision rate of the model before and after the improvement was relatively close, and both of them began to converge in about 80 groups. However, it was impossible to judge the prediction effect of a model only from the precision rate. In **Figure 13B**, the models before and after the improvement began to converge in about 60 groups, but the recall of the improved YOLOv5 model was always greater than that of the YOLOv5 model. **Figurse 13C, D** showed that, in the YOLOv5 model, when the recall rate was less than 0.45, the precision rate remained at 1. In the improved YOLOv5 model, when the recall rate was less than 0.55, the precision rate remained at 1. For the YOLOv5 model, the inflection point of P-R curve appears before the recall rate was 0.8, while for the improved YOLOv5 model, the inflection point of P-R curve appears after the recall rate was 0.8, and the P-R curve was smoother. This was because when the recall rate became larger, the advantage of the model with CA attention mechanism module became obvious and the learning ability became stronger. The original model thought that the contribution of each region in the images was evenly distributed, but in the actual detection process, affected by the size, color and occlusion of the pods, the model had different and complex regions of interest for different images. The model after embedding the attention mechanism focused on the information useful for the detection category, and the use of EIOU Loss frame regression loss function took into account the real difference between the width and height of the pods and the confidence, so that the occluded pods were not easy to be incorrectly detected or missed. In addition, the average precision of pods recognition of the YOLOv5 model was 88.7%, and that of the improved YOLOv5 model was 91.7%. The mAP value of the improved model was increased by 3%. The results showed that the improved model had strong generalization ability and higher recognition accuracy for pods in different growth stages.

#### 4.1.3 Performance evaluation of pod recognition model

In this paper, the performance of the traditional YOLOv5 and the improved YOLOv5 models for pod’s recognition was compared and analyzed by using four indicators: detection rate (Xu Z. et al., 2022), test time, calculation amount (FLOPs) required for processing an image, and parameter amount (Hsia et al., 2021). **Table 3** showed the comparison of pods recognition performance of different models in the data set.

**Table 3:** Comparison of performance evaluation indexes of different models.

Model	Model performance evaluation index			
	FPS (frame /ms)	Test time/ms	FLOPs	Parameter quantity
YOLOv5	18.18	44.258	4.79×10^10^	2.845×10^7^
Improved YOLOv5	24.39	32.759	3.95×10^10^	2.085×10^7^

FPS referred to the number of images that the model could process per millisecond. The larger the FPS, the higher the rate of the model and the better the performance. Test time referred to the time taken to process all images in the testing set, including pre-processing time, network pre-processing time and post-processing time. The calculation quantity and parameters quantity of the model reflected the complexity of the model. **Table 3** showed that the improved YOLOv5 model could process 24.39 pictures per second, the test time of the model was 32.759s, and the calculation amount and parameter amount of the model were 4.79×10^9^ and 2.085×10^7^, respectively. Compared with the original YOLOv5 model, the detection rate was increased by 34.16%, the test time was saved by 11.499s. The calculation amount and parameter amount of the model were reduced by 17% and 7.6%, respectively. From the above analysis, it could be seen that the improved YOLOv5 model, which embedded CA module and EIOU regression loss function, could not only reduce the amount of calculation and parameters of the model, but also improved the accuracy of pods recognition.

### 4.2 Results analysis of soybean yield estimation

#### 4.2.1 Result analysis of pod’s phenotype calculation

**Figure 14:**
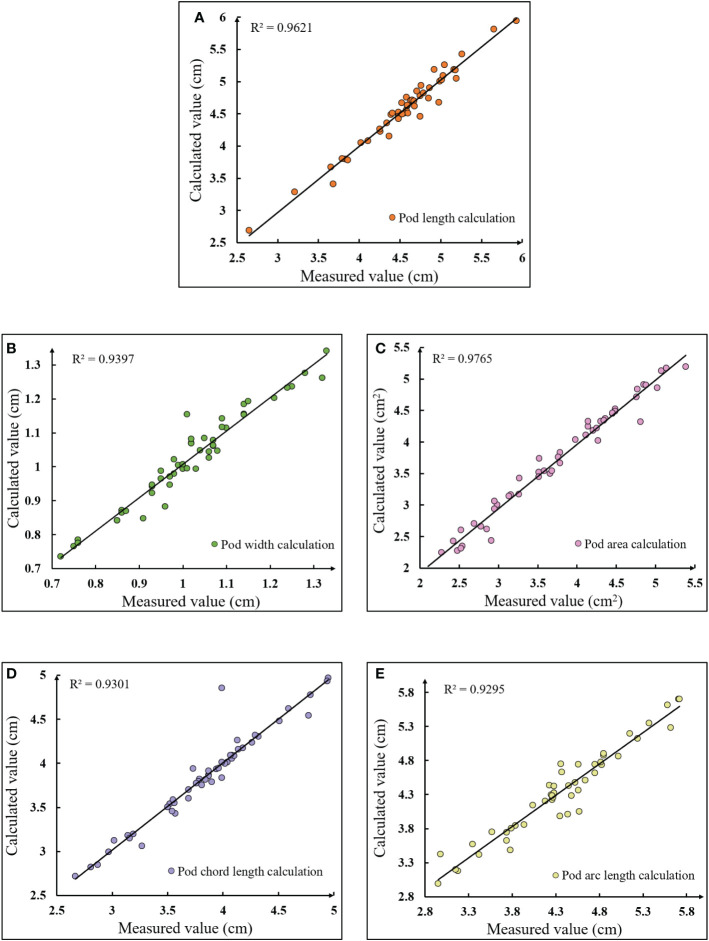
Linear correlation between calculated and measured values of pods phenotypic traits **(A)** Pod length calculation; **(B)** Pod width calculation; **(C)** Pod area calculation; **(D)** Pod chord length calculation; **(E)** Pod arc length calculation.


The actual manual measurement value of electronic vernier caliper was used as the standard value of pod’s length, width and chord length. The number of pixels counted by Photoshop tool (PhotoshopCC2017, San Jose, US) was used as the standard value of the pod’s area and convex arc length. In order to verify the effectiveness of the calculation method of phenotypic traits for soybean pods, Equations (24-26) were used to calculate the relative error, average absolute error and average relative error between the calculated and measured values. The correlation between the calculated and measured values of pod length, pod width, pod area, chord length and arc length were shown in **Figure 14**.

(24) $$P_{\text{i}}=\frac{\left|x_{i}-\bar{x_{i}}\right|}{\bar{x_{i}}}\times 100\%$$

(25) $$AE=\frac{1}{m}\sum_{i=1}^{m}\left|x_{i}-\bar{x_{i}}\right|$$

(26) $$RE=\frac{1}{m}\sum_{i=1}^{m}p_{i}\times 100\%$$

Where, *p_i_* represented the relative error in the calculation of phenotypic traits of a single pod; *AE* represented the mean absolute error in the calculation of phenotypic traits of a single pod; *RE* represented the average relative error in the calculation of phenotypic traits of a single pod; *x_i_* represented the calculated value of phenotypic traits of a single pod; $\bar{x_{i}}$ represented the measured value of phenotypic traits of a single pod; m represented the number of pods.

**Figures 14A–E** showed the linear correlation between the calculated and measured values of pod length, pod width, pod area, chord length and arc length at maturity, with coefficients of determination of 0.962, 0.939, 0.976, 0.930 and 0.929, respectively. It could be seen from the figure that the length of pods at maturity was mainly distributed between 4.5~5.5cm, the width was mainly distributed between 0.95~1.2cm, the area was mainly distributed between 2.5~5cm^2^, and the chord length and arc length were mainly distributed between 3.5~4.5cm and 3.8~5.3cm. According to Equation (25), the average absolute errors of pod length, width, area, chord length and arc length were 0.084cm, 0.025cm, 0.096cm^2^, 0.063cm and 0.12cm, respectively; According to Equation (26), the average relative errors were 1.85%, 2.46%, 2.83%, 1.65% and 2.86%, respectively. It could be seen that there was an obvious linear correlation between the calculated value and the measured value obtained by the pod’s phenotypic traits calculation method. The average coefficient of determination R^2^ was 0.947, and the average relative error was 2.33%. Thus, the phenotypic traits of pods could be calculated quickly and accurately by using the proposed methods.

#### 4.2.2 Result analysis of soybean yield estimation

##### 4.2.2.1 (1) Result analysis of estimating the weight of a single pod

**Figure 15:**
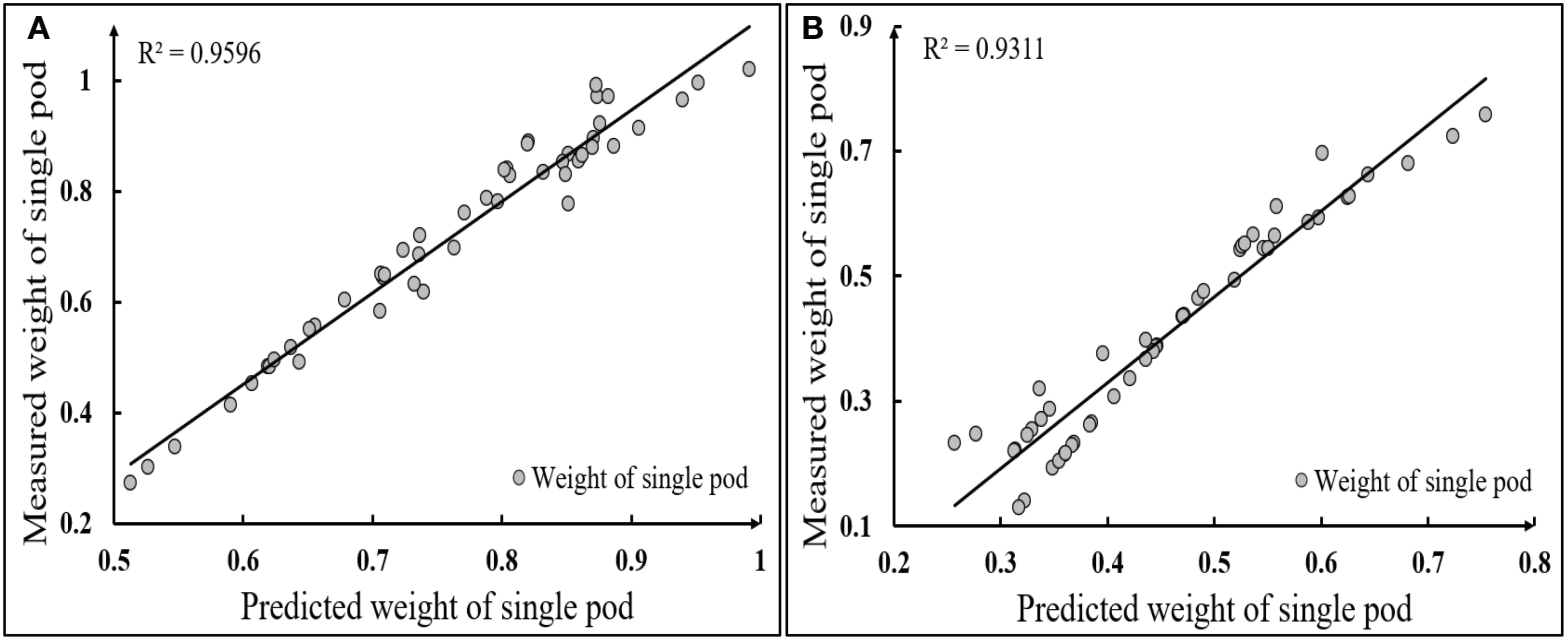
Linear correlation between estimated and measured values of single pod weight **(A)** Heihe 49; **(B)** Suinong 26.

In this study, 100 soybean pods of two varieties at R8 stage were trained using the proposed single pod weight estimation model. The training results were shown in **Table 4**. The pod’s weight measured by the laboratory electronic weighing scale with an accuracy of 0.0001g was used as the standard value, and the pod’s weight obtained by model training was used as the prediction value. The linear correlation between the predicted value of a single pod and the measured value was shown in **Figure 15**.

**Table 4:** Statistics of pods weight estimation model at maturity stage.

Varieties	Average value of pods phenotypic traits (cm)					Estimated weight (g)	Actual weight (g)	MSE
	Pod length	Pod width	Pod area	Chord length	Arc length			
Heihe 49	4.56	1.02	4.11	3.85	4.34	0.726	0.767	0.0089
Suinong 26	4.20	0.98	3.70	4.14	4.43	0.409	0.458	0.0084

**Table 4** showed that the estimated average weight of single pod of Heihe 49 and Suinong 26 was 0.726g and 0.409g, respectively, which was 0.041g and 0.049g lower than the actual value measured by the electronic libra with an accuracy of 0.0001g. The MSE reflected the difference between the actual value and the estimated value. The MSE of the two varieties of model training was 0.0089 and 0.0084, respectively. **Figure 15** showed that the coefficient of determination R^2^ between the estimated and measured single pod’s weight of Heihe 49 and Suinong 26 was 0.9596 and 0.9311, respectively, and the single pod’s weight of Heihe 49 was mainly distributed between 0.6 and 0.9g, while that of Suinong 26 was mainly distributed between 0.3 and 0.7g. From the above analysis, it could be seen that the average of the MSE of the estimation of the single pod weight of the two varieties was 0.00865, and the average coefficient of determination between the estimated weight and the actual weight was 0.9453. Thus, the type of 5-120-1 BP neural network constructed in this study was effective and stable for the prediction of the pods’ weight.

##### 4.2.2.2 (2) Result analysis of estimating the weight of the whole plant pods

Three pots of soybean were randomly selected from Heihe 49 and Suinong 26. By combining the average number of pods per plant recognized by the improved YOLOv5 algorithm with the average weight of a single pod estimated in result analysis of pod’s phenotype calculation, and the pods weight of all the potted soybeans plants of this variety could be predicted. **Table 5** showed the prediction information of potted soybean yield.

**Table 5:** Prediction information of potted soybean yield.

Varieties	Weight of single pod (g)	Number of pods per plant	Estimation of pods weight per plant (g)	Predicted weight of all pods (g)	Measured weight of all pods (g)	Relative error
Hehei 49	0.726	19	13.794	165.58	149.60	0.106
Suinong 26	0.409	28	11.452	1076.49	945.70	0.138

**Table 5** showed that combining the pod’s number per plant estimated by the proposed method with the weight of a single pod. The predicted total weight of all potted soybeans of Heihe 49 and Suinong 26 were 165.58g and 1076.49g, respectively. According to Equation (24), the relative errors of pods weight prediction of Heihe 49 and Suinong 26 were 0.106 and 0.138 respectively. The relative error of pods weight prediction of Heihe 49 was 0.032, lower than that of Suinong 26, which mainly because Heihe 49 had shorter stems than Suinong 26 at maturity stage, and the degree of shielding between pods, pods and stems was smaller, so the accuracy of pods number detection was higher. According to the above analysis, the average relative error of pods weight prediction of the two varieties of soybean was 0.122. It could be seen that the average value calculated by the phenotypic traits of a single plant was effective for the yield prediction of all potted soybean plants which could provide a technical reference for the yield prediction of soybean plants in the field and the breeding of excellent soybean varieties.

## 5 Discussion

(1) Comparative analysis of related studies

The counting of pods and the estimation of pods weight per plant are of great significance for the breeding, cultivation and field management of soybean varieties. Due to the successful application of machine vision technology in the field of agricultural phenotype exploration, researchers began to use these technologies to obtain soybean phenotypes in high-throughput and high-precision, so as to speed up crop improvement and breeding of new varieties to achieve high yield of soybean plants. Guo, et al improved the YOLOv4 target detection algorithm (Guo et al., 2021). For mature soybean plants without leaves, the number of pods per plant and the number of seeds in pods were detected. In this study, the coordinate attention mechanism was combined with the traditional YOLOv5 model to achieve the accurate recognition of pods in complex images with leaf occlusion under the outdoor growth state. Compared with the literature (Ning et al., 2021), the recognition accuracy of the number of pods in this study has been improved by 5.46%, and the prediction model of soybean yield has been constructed by combining BP neural network. Compared with the calculation methods of soybean pod length, width, area and other phenotypic traits proposed in the literature (Uzal et al., 2018; Yan et al., 2020; Li et al., 2021; Yang et al., 2022), the intelligent calculation methods used in this study not only had higher accuracy and less calculation, but also do not require manual marking, avoiding the error caused by human subjective judgment effectively.

(2) Establishment of pods recognition model

In this paper, an improved YOLOv5 model was proposed, which realized the accurate recognition of pods in different growth stages, and the recognition accuracy rate reached 91.7%. It solved the problems of mutual occlusion, unclear edges and difficult detection of small target pods in the process of soybean pods recognition. However, from the actual test results, it was found that a few pods were not correctly detected due to serious occlusion or similar shape and color to the leaves. In view of this phenomenon, the acquisition equipment with higher resolution should be used to obtain the soybean plant images, and the soybean plants should be rotated for several times to shoot, and the images with the least occlusion should be selected. At the same time, we should further improve the model feature extraction network from the internal structure of the network, so that the model paid more attention to the color, shape and texture features of pods, and improved the accuracy of pods detection.

(3) Error analysis

The average errors of soybean pod’s recognition and yield prediction methods based on the improved depth learning model proposed in this paper were 8.3% and 12.2%, respectively. On the one hand, the error was caused by the algorithm itself. In the detection process, a few pods will be missed or wrongly detected. On the other hand, it was the geometric distortion caused by the machine vision system and shooting angle when acquiring images, and the cumulative error caused by the precision limitation of the instrument itself when measuring. In this regard, the acquisition equipment with higher resolution should be used and the camera position should be fixed to make it perpendicular to the object to be measured and maintain a fixed distance during the shooting process to reduce the geometric distortion caused by the raw image acquisition. For the accuracy limitation of the measuring instrument itself and the error in measurement, the average value of multiple measurements should be taken as the measured value to reduce the error.

(4) Application and promotion

In this study, CA attention mechanism was combined with YOLOv5 algorithm to construct a lightweight depth learning method, which was applied to the detection of pods in different growth stages successfully. The model had the advantages of high precision, small scale, less parameters, and greatly reduced the calculation amount of the model, which can meet the deployment requirements of portable mobile terminal devices. The future work should be based on the research content of this paper, combine the computer vision technology with the research of crop phenotypic parameters, and achieve accurate prediction of soybean yield in the field environment. For the method of narrow row and dense planting, high resolution color image sensor carried by Unmanned Aerial Vehicle (UAV) can be used to obtain multi angle soybean plant images. For the method of three ridge planting, the movable platform can be directly used to take the soybean plant images at the same time interval from different angles, and different degree of overlapping of pods, etc. Finally, recognition algorithms will be optimized to select images that best reflects the actual situation of the pods from the acquired images for this research.

## 6 Conclusion

In this study, a soybean pods recognition method based on improved YOLOv5 algorithm in different growth stages was proposed, and the prediction of soybean yield was realized by combining the pod phenotypic traits obtained by intelligent calculation methods. The experimental results showed that, the average accuracy of the proposed model reached 91.7%, increasing by 3% compared with traditional YOLOv5 model. The coefficients of determination R^2^ between the calculated value and the measured value of pod’s length, width, area, chord length and convex edge arc length were 0.962, 0.939, 0.976, 0.930 and 0.929, respectively. The MSE of single pod weight prediction was 0.00865, and the average coefficients of determination between the predicted value and the actual value was 0.945. Combined with the detection of pods per plant by the improved model, the MRE of all potted soybean yield predictions was 0.122. The proposed methods not only achieved high-precision recognition of pods and calculation of phenotypic traits, but also provided quantitative basis and technical support for estimation of soybean yield and cultivation of excellent soybean varieties in agronomy.

## Data availability statement

The original contributions presented in the study are included in the article/Supplementary Material. Further inquiries can be directed to the corresponding author.

## Author contributions

XM and HG conceived and designed the experiments. HH, PS and FW performed the experiments and acquired the digital image data of soybean plants in different growth stages. HH and HG analyzed and processed the data. HH and XM wrote the paper. All authors contributed to the article and approved the submitted version.
